# *Naevus Maternus*, Mother's Mark

**Published:** 1868-02

**Authors:** 


*Naevus Maternus*, Mother's mark.—A mark or spot on the skin of children when born, presenting a variety of appearances and attributed to the influence of the maternal imagination.

Thomas Smith F.R.C.S., of St. Bartholomew's Hospital, London, in *Clinical Papers on the Surgery of Childhood*, says that "there are many well authenticated cases where marks and even bodily deformities in the fœtus can be fairly attributed to strong and persistent mental impressions in the mother;" and describes the following striking case of a child admitted into St. Bartholomew's Hospital in 1865. "She was at that time twelve years old. The left upper extremity and the greater part of the corresponding side of the trunk and neck were deeply stained with dark-brown pigment, from which grew an abundant crop of brown, harsh, lank hair, varying in length from one to two inches. The skin was rough and harsh; the arm was long, thin and withered; the scapula was unnaturally prominent. In fact the upper limb, shoulder, and back bore a very strong resemblance to the corresponding part of a monkey. The mother stated that when she was three months pregnant with the child, she was much terrified by a monkey attached to a street organ, which jumped on her back as she was passing by."

The same writer speaking of the treatment necessary for the removal of moles says that the only plan is by complete removal either with the knife or by some kind of cautery. As a caustic, he recommends the strong nitric acid as the most manageable and efficient means of cure, and the only one that has given him the best results. He also remarks that he need scarcely say that in infants the only moles that require treatment are such as by their size or position are manifest blemishes.

